# Intravitreal aflibercept 8 mg in patients from Japan with diabetic macular edema: 48-week subgroup analysis of the PHOTON trial

**DOI:** 10.1007/s10384-025-01271-7

**Published:** 2025-12-26

**Authors:** Kiyoshi Suzuma, Toshinori Murata, Masahiko Shimura, Shigeo Yoshida, Genichiro Kishino, Alyson J. Berliner, Karen W. Chu, Kimberly Reed, Robert Vitti, Yenchieh Cheng, Delia Voronca, Rafia Bhore, Sergio Leal, Peter Morgan-Warren, Andrea Schulze, Ursula Schmidt-Ott, Masato Kobayashi, Taiji Sakamoto

**Affiliations:** 1 Department of Ophthalmology, Kagawa University Faculty of Medicine, Kagawa University Hospital, Kita-gun, Kagawa, Japan; 2 Department of Ophthalmology, Shinshu University School of Medicine, Shinshu University Hospital, Matsumoto, Nagano Japan; 3 Department of Ophthalmology, Tokyo Medical University Hachioji Medical Center, Hachioji, Tokyo, Japan; 4 Department of Ophthalmology, Kurume University School of Medicine, Kurume, Fukuoka Japan; 5 Department of Ophthalmology, Kozawa Eye Hospital and Diabetes Center, Mito, Ibaraki Japan; 6 Regeneron Pharmaceuticals, Inc., Tarrytown, NY USA; 7 Bayer Consumer Care AG, Basel, Switzerland; 8 Bayer AG, Berlin, Germany; 9 Bayer Yakuhin, Ltd., Osaka, Japan; 10 Department of Ophthalmology, Kagoshima University, Kagoshima, Japan

**Keywords:** Aflibercept, Diabetic macular edema, Diabetic retinopathy, Anti-VEGF agent, Vascular endothelial growth factor

## Abstract

**Purpose:**

In the pivotal PHOTON trial of patients with diabetic macular edema (DME), aflibercept 8 mg administered every 12 (8q12) and 16 (8q16) weeks demonstrated similar visual and anatomic outcomes with no new safety signals to aflibercept 2 mg every 8 weeks (2q8). We conducted a prespecified subgroup analysis to assess the efficacy, durability, and safety of aflibercept 8 mg in the Japanese patients from PHOTON.

**Study design:**

Prespecified subgroup analysis of the Phase 3 PHOTON trial (NCT04429503).

**Methods:**

Adult patients with DME were randomized 1:2:1 to receive intravitreal aflibercept 2q8, 8q12, or 8q16 following initial monthly doses. Patients randomized to 8q12 and 8q16 were eligible for dose regimen modification. The primary endpoint was change from baseline in best-corrected visual acuity (BCVA) at Week 48. Prespecified efficacy and safety outcomes at/through Week 48 are reported, segmented by Japan versus the rest of world (non-Japan).

**Results:**

In the Japan and non-Japan subgroups, respectively, mean changes in BCVA were +7.0 and +9.0 (8q12), +7.4 and +7.9 (8q16), and +8.0 and +9.4 (2q8) letters at Week 48; differences in least squares means were -0.30 and -0.64 letters between 8q12 and 2q8 and +0.17 and -1.76 letters between 8q16 and 2q8; ocular treatment-emergent adverse events were reported in 32.4% and 31.6% (8q12), 35.3% and 28.8% (8q16), and 30.0% and 27.2% (2q8) of patients.

**Conclusion:**

Improvements in BCVA at Week 48 were generally similar with aflibercept 8 mg versus 2 mg in this subgroup analysis of Japanese and non-Japanese patients with DME, suggesting that the primary findings from PHOTON may be generalized to the Japanese population.

**Supplementary Information:**

The online version contains supplementary material available at 10.1007/s10384-025-01271-7.

## Introduction

Diabetic retinopathy (DR) is a microvascular complication of diabetes mellitus and a major cause of vision loss in the working-age population [1]. As the prevalence of diabetes increases worldwide [2], the number of adults with diabetic macular edema (DME)—a vision-threatening complication of DR—is also projected to increase, with an estimated global prevalence of 160.5 million by 2045 [3]. This increase is likely to substantially impact the Japanese population, as evidenced by the increasing prevalence of DME from a previous claims analysis of Japanese individuals [4].

Vascular endothelial growth factor (VEGF) plays a central role in the pathogenesis of DR [5]. Hyperglycemia in diabetes leads to microvascular abnormalities, resulting in decreased perfusion and increased VEGF signaling, which can lead to the development of DR [5]. Given that VEGF is a key driver of vascular permeability, excessive VEGF signaling can lead to breakdown of the blood-retinal barrier and subsequent fluid accumulation in the macula, resulting in DME [5, 6].

Intravitreal anti-VEGF therapy has revolutionized the treatment of center-involved DME and is regarded as first-line therapy [7, 8]. Data from pivotal trials with intravitreal anti-VEGF agents have consistently demonstrated efficacy and safety for the treatment of DME [9–14]. The intravitreal anti-VEGF agent aflibercept is a recombinant fusion protein that acts as a soluble decoy receptor by binding VEGF-A and placental growth factor and inhibiting activation of cognate receptors [15, 16]. Efficacy and safety of aflibercept 2 mg in DME was initially demonstrated in the phase 3 VISTA and VIVID trials, in which treatment markedly improved visual and anatomic outcomes compared with laser photocoagulation [9–11]. The clinical efficacy and safety of aflibercept 2 mg in Japanese patients with DME was further evaluated in the VIVID-Japan trial, which also demonstrates substantial improvements in visual and anatomic outcomes in patients treated with aflibercept 2 mg versus laser photocoagulation [17].

Maintenance of visual and anatomic improvements with intravitreal anti-VEGF therapies is often challenging in clinical practice due to frequent injection and monitoring schedules that lead to non-adherence and suboptimal treatment outcomes [18–21]. There is, therefore, an unmet need for treatments with extended durability that support improved treatment outcomes in patients with DME [22].

Aflibercept 8 mg was designed to prolong the suppression of VEGF signaling [23]. In the pivotal PHOTON trial in patients with DME, aflibercept 8 mg administered every 12 and 16 weeks (8q12 and 8q16, respectively) demonstrated non-inferiority in best-corrected visual acuity (BCVA) gains at a 4-letter margin compared to aflibercept 2 mg administered every 8 weeks (2q8) at Week 48 [24]. Furthermore, treatment with aflibercept 8 mg allowed for extended dosing intervals in the majority of patients (91% of patients in the 8q12 group and 89% of patients in the 8q16 group maintained 12- and 16-week dosing intervals, respectively), with similar visual and anatomic outcomes and no new safety signals compared to aflibercept 2q8 [24]. Importantly, findings from the PHOTON trial supported regulatory approval of aflibercept 8 mg for the treatment of DME in several countries including Japan, the United Kingdom, those of the European Union, and the United States, where it is also approved for DR [15, 25–27].

Herein, we report a 48-week subgroup analysis of the PHOTON trial to assess the efficacy, durability, and safety of aflibercept 8 mg in Japanese patients with DME.

## Subjects and methods

### Study design

The design of the PHOTON trial (ClinicalTrials.gov identifier: NCT04429503) has been previously described [24]. Briefly, PHOTON was a randomized, active-controlled, double-masked, 96-week, non-inferiority, phase 2/3 trial conducted across 138 sites in seven countries (Canada, Czechia, Germany, Hungary, Japan, the United Kingdom, and the United States). The trial was conducted in accordance with the International Council for Harmonisation E6 Guideline for Good Clinical Practice, the principles of the Declaration of Helsinki, and all applicable local, state, and federal laws. The trial protocol and amendments were approved by an institutional review board and ethics committee before trial initiation (Online Resource 1). All patients provided written informed consent prior to participation in the trial.

The PHOTON trial enrolled patients aged 18 years or older with type 1 or type 2 diabetes and DME with central involvement (central retinal thickness [CRT] ≥ 300 µm) as determined by the reading center at screening, with one eye per patient designated as the study eye [24]. Eligible patients were also required to have a BCVA score of 78 to 24 Early Treatment Diabetic Retinopathy Study (ETDRS) letters (approximate Snellen equivalent 20/32 to 20/320) in the study eye, with decreased vision primarily attributed to DME. Patients were excluded from the trial if they exhibited macular edema due to any cause other than diabetes; had active proliferative DR; or if they had had panretinal laser photocoagulation, macular laser photocoagulation, or intravitreal anti-VEGF treatment in the study eye within 12 weeks of screening. Full inclusion and exclusion criteria have been previously described [24].

Eligible patients were randomized 1:2:1 to receive intravitreal aflibercept 2q8 following five initial monthly doses, or aflibercept 8q12 or 8q16 following three initial monthly doses through Week 96 [24]. Beginning at Week 16, patients randomized to the 8q12 and 8q16 groups who met the protocol-specified criteria were eligible for dose regimen modification (DRM); dosing intervals for patients treated with aflibercept 8 mg were shortened if they experienced a > 10-letter loss in BCVA from Week 12 due to persistent or worsening DME and a > 50-µm increase in CRT from Week 12, as previously described [24]. Patients in the 8q12 and 8q16 groups who met DRM criteria at Weeks 16 or 20 had their dosing intervals shortened to every 8 weeks, and those who met DRM criteria after Week 24 had their dosing intervals shortened by 4 weeks. The minimum dosing interval was 8 weeks, and no extension of dosing intervals was permitted in Year 1.

As previously described [24], patients were monitored every 4 weeks over the duration of the study. Key ocular assessments at prespecified timepoints included BCVA (using the ETDRS BCVA protocol), spectral-domain optical coherence tomography, intraocular pressure (IOP), and color fundus photography.

### Outcomes

Efficacy and safety endpoints presented in this subgroup report are analogous to the prespecified endpoints in the 48-week analysis of the PHOTON trial [24]. The primary endpoint was change from baseline in BCVA at Week 48; a key secondary endpoint was the proportion of patients with ≥ 2-step improvement from baseline in Diabetic Retinopathy Severity Scale (DRSS) score at Week 48. Change from baseline in CRT at Week 48 was assessed as an additional secondary endpoint, and the proportion of patients randomized to 8q12 or 8q16 that maintained their randomized dosing intervals through Week 48 was assessed as an exploratory endpoint. Incidence of ocular and non-ocular treatment-emergent adverse events (TEAEs) and serious TEAEs through Week 48 was also evaluated.

### Statistical analysis

This report summarizes data through Week 48 (database lock on August 19, 2022) for all randomized patients segmented by Japan versus the rest of the world (non-Japan). Efficacy and safety analyses were conducted using the same statistical analysis methods previously described for the overall PHOTON population [24]. Efficacy analyses were conducted in the full analysis set (FAS), which included all randomized patients who received at least one dose of study treatment and were based on treatment assigned at randomization irrespective of any shortening of dose interval. Safety analyses were conducted in the safety analysis set (SAF), which included all randomized patients who received at least one dose of study treatment, and were based on actual treatment received irrespective of any shortening of dose interval. Durability for aflibercept 8 mg-treated patients was analyzed using the SAF that completed Week 48. All data were summarized descriptively.

The analysis of the primary efficacy endpoint, using differences in least squares (LS) mean change from baseline in BCVA at Week 48 between the 8q12 and 2q8 groups and between the 8q16 and 2q8 groups, was based on a mixed model for repeated measures that included baseline BCVA as the covariate; treatment group, visit, stratification variables (geographic region [Japan vs. rest of world], baseline CRT [< 400 μm vs. ≥ 400 μm], and previous treatment for DME [yes vs. no]) as fixed factors; as well as terms for the interaction between baseline BCVA and visit and for the interaction between treatment and visit.

The analysis of the key secondary endpoint, using differences in the proportion of patients with ≥ 2-step improvement from baseline in DRSS score at Week 48 between the 8q12 and 2q8 groups, and between the 8q16 and 2q8 groups, was based on the Cochran-Mantel-Haenszel (CMH) test stratified by stratification variables, with last observation carried forward for patients with missing DRSS scores at Week 48. Differences in the proportions are reported with two-sided 95% CMH confidence intervals (CIs) that were calculated using normal approximation.

Intercurrent events and missing data were handled as previously described for both the primary and key secondary endpoints [24]. Additional secondary and exploratory endpoints in the Japan and non-Japan subgroups were analyzed using the same methodology as previously described [24]. Safety data were summarized descriptively. Ocular and non-ocular TEAEs were coded using the Medical Dictionary for Regulatory Activities version 25.0.

## Results

### Patients

As previously described [24], 660 patients were enrolled in PHOTON and randomized to receive aflibercept 8q12 (n = 329), 8q16 (n = 164), or 2q8 (n = 167), with 658 patients included in the FAS/SAF (8q12: n = 328; 8q16: n = 163; 2q8: n = 167). The Japan subgroup (FAS/SAF) comprised 74 patients (8q12: n = 37; 8q16: n = 17; 2q8: n = 20) and the non-Japan subgroup (FAS/SAF) comprised 584 patients (8q12: n = 291; 8q16: n = 146; 2q8: n = 147; Online Resource 2).

Baseline demographic and ocular characteristics were generally balanced across treatment groups (Table 1). In the Japan subgroup, mean age at baseline was 64.0 years (vs. 62.1 years in the non-Japan subgroup), 64.9% (48/74) of patients were men (vs. 60.4% [353/584] of patients in the non-Japan subgroup), and 100% (74/74) of patients were Asian (vs. 4.6% [27/584] of patients in the non-Japan subgroup). In the Japan subgroup, 27.0% (10/37) of patients in the 8q12 group had a DRSS level of 47 or worse, compared with 23.5% (4/17) in the 8q16 group and 30.0% (6/20) in the 2q8 group. In the non-Japan subgroup, 35.4% (103/291) of patients in the 8q12 group had a DRSS level of 47 or worse, compared with 28.8% (42/146) in the 8q16 group and 32.0% (47/147) in the 2q8 group. In the Japan subgroup, mean BCVA was 58.2 in the 2q8 group, while the 8q12 and 8q16 groups were approximately one line better (63.2 and 62.0 letters, respectively). Across all treatment groups, 55.4% (41/74) of patients in the Japan subgroup and 42.3% (247/584) of patients in the non-Japan subgroup were previously treated for DME. Notably, mean hemoglobin A1c (7.4 vs. 8.0) and the proportion of patients with hypertension (50.0% [37/74)] vs. 81.7% [477/584]) was lower in the Japan subgroup versus the non-Japan subgroup, respectively.

**Table 1:** Baseline demographics and characteristics for the Japan and non-Japan subgroups in PHOTON

	Japan				Non-Japan			
	Aflibercept 2q8 (n = 20)	Aflibercept 8q12 (n = 37)	Aflibercept 8q16 (n = 17)	Total (N = 74)	Aflibercept 2q8 (n = 147)	Aflibercept 8q12 (n = 291)	Aflibercept 8q16 (n = 146)	Total (N = 584)
Age (years), mean (SD)	65.9 (11.1)	62.5 (13.9)	65.1 (11.9)	64.0 (12.7)	62.7 (9. 6)	62.0 (10.8)	61.5 (9.2)	62.1 (10.1)
Sex, n (%)								
Male	12 (60.0)	27 (73.0)	9 (52.9)	48 (64.9)	80 (54.4)	183 (62.9)	90 (61.6)	353 (60.4)
Female	8 (40.0)	10 (27.0)	8 (47.1)	26 (35.1)	67 (45.6)	108 (37.1)	56 (38.4)	231 (39.6)
Race or ethnicity, n (%)								
White	0	0	0	0	112 (76.2)	231 (79.4)	128 (87.7)	471 (80.7)
Asian	20 (100)	37 (100)	17 (100)	74 (100)	10 (6.8)	11 (3.8)	6 (4.1)	27 (4.6)
Black or African American	0	0	0	0	18 (12.2)	35 (12.0)	9 (6.2)	62 (10.6)
Other^a^	0	0	0	0	4 (2.7)	10 (3.4)	1 (0.7)	15 (2.6)
Hispanic or Latino	0	0	0	0	31 (21.1)	54 (18.6)	34 (23.3)	119 (20.4)
Non-ocular characteristics								
HbA1c (%), mean (SD)	7.3 (0.7)	7.4 (1.0)	7.4 (0.7)	7.4 (0.9)	8.3 (1.5)	8.0 (1.6)	7.9 (1. 6)	8.0 (1.6)
Type 2 diabetes, n (%)	19 (95.0)	34 (91.9)	16 (94.1)	69 (93.2)	137 (93.2)	276 (94.8)	138 (94.5)	551 (94.3)
Hypertension, n (%)	12 (60.0)	16 (43.2)	9 (52.9)	37 (50.0)	118 (80.3)	238 (81.8)	121 (82.9)	477 (81.7)
Duration of diabetes (years), mean (SD)	15.3 (12.1)	12.3 (10.3)	10.7 (9.4)	12.7 (10.6)	16.0 (9.8)	15.5 (9.9)	16.3 (10.7)	15.8 (10.1)
Ocular characteristics								
BCVA (ETDRS letters), mean (SD)	58.2 (12.4)	63.2 (9.6)	62.0 (12.2)	61.6 (11.1)	61.9 (11.0)	63.7 (10.2)	61.4 (11.8)	62.7 (10.8)
CRT (µm), mean (SD)	454.2 (120.8)	446.1 (108.2)	453.9 (104.2)	450.1 (109.4)	457.7 (147.2)	449.5 (129.8)	461.1 (119.6)	454.5 (131.9)
DRSS status, n (%)^b^								
DR absent or questionable; mild-to-moderate NPDR (DRSS level 10, 12, 14, 15, 20, 35, 43)	14 (70.0)	26 (70.3)	12 (70.6)	52 (70.3)	91 (61.9)	171 (58.8)	95 (65.1)	357 (61.1)
Moderately severe-to-severe NPDR (DRSS level 47, 53)	3 (15.0)	7 (18.9)	3 (17.6)	13 (17.6)	36 (24.5)	73 (25.1)	23 (15.8)	132 (22.6)
PDR (DRSS level 61, 65, 71, 75)	3 (15.0)	3 (8.1)	1 (5.9)	7 (9.5)	11 (7.5)	30 (10.3)	19 (13.0)	60 (10.3)
Missing/ungradable (DRSS level 90)	0	1 (2.7)	1 (5.9)	2 (2.7)	9 (6.1)	17 (5.8)	9 (6.2)	35 (6.0)
Prior DME treatment, n (%)								
Treatment naive	9 (45.0)	16 (43.2)	8 (47.1)	33 (44.6)	84 (57.1)	169 (58.1)	84 (57.5)	337 (57.7)
Previously treated	11 (55.0)	21 (56.8)	9 (52.9)	41 (55.4)	63 (42.9)	122 (41.9)	62 (42.5)	247 (42.3)

Full analysis set. Percentages are based on n values provided in the column headings

*2q8* aflibercept 2 mg every 8 weeks, *8q12* aflibercept 8 mg every 12 weeks, *8q16* aflibercept 8 mg every 16 weeks, *BCVA* best-corrected visual acuity, *CRT* central retinal thickness, *DME* diabetic macular edema, *DR* diabetic retinopathy, *DRSS* Diabetic Retinopathy Severity Scale, *ETDRS* Early Treatment Diabetic Retinopathy Study, *HbA1c* hemoglobin A1c, *NPDR* non-proliferative diabetic retinopathy, *PDR* proliferative diabetic retinopathy, *SD* standard deviation

^a^Other includes patients who were American Indian or Alaska Native, Native Hawaiian or Other Pacific Islander, multiracial, or other

^b^Level 10, 12, none; levels 14, 15, 20, DR questionable; level 35, mild NPDR; level 43, moderate NPDR; levels 47, 53, moderately severe-to-severe NPDR; level 61, mild PDR; level 65, moderate PDR; levels 71 and 75, high-risk PDR

Patients in the Japan subgroup who had completed Week 48 received a mean of 6.1, 5.0, and 8.0 injections in the 8q12, 8q16, and 2q8 groups, respectively. The corresponding mean number of injections for patients in the non-Japan subgroup was 5.9, 5.0, and 7.9.

### BCVA

**Fig. 1:**
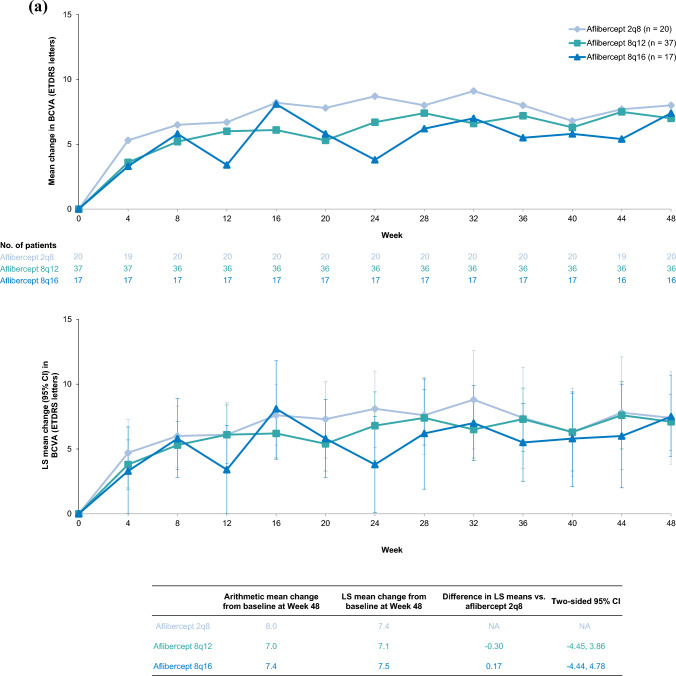

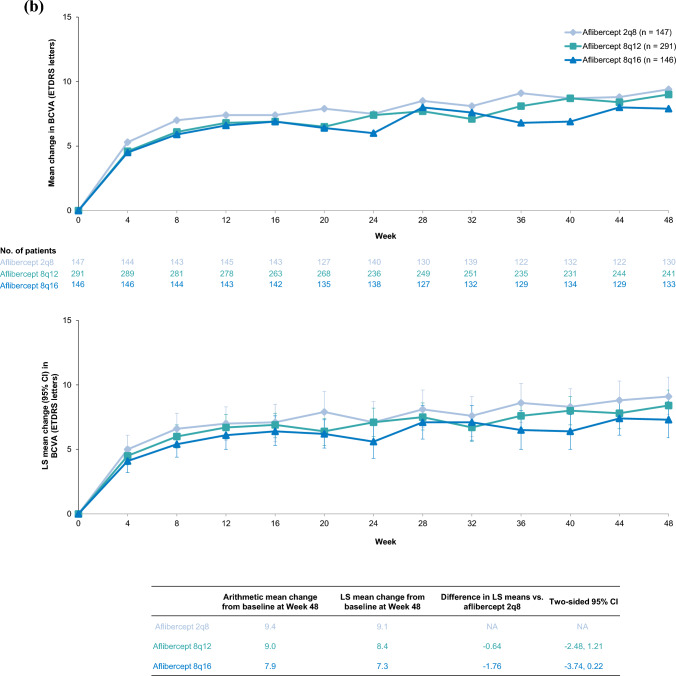
Arithmetic mean change and LS mean change from baseline in BCVA through Week 48 in the (a) Japan and (b) non-Japan subgroups of PHOTON. Full analysis set. Mean change from baseline in BCVA through Week 48 in the Japan subgroup (**a**) and the non-Japan subgroup (**b**) are presented in the top panels. Data are arithmetic means of observed data before relevant intercurrent events, as previously defined [24]. The number of patients included in the analysis, after accounting for relevant intercurrent events and missing data, are shown for each timepoint. LS mean change from baseline in BCVA at Week 48 in the Japan subgroup (**a**) and the non-Japan subgroup (**b**) are presented in the bottom panels. LS means were generated using MMRM, based on observed data before relevant intercurrent events, with baseline BCVA as a covariate; treatment group, visit, stratification variables (geographic region [Japan vs. rest of world], baseline CRT [< 400 μm vs. ≥ 400 μm], and previous treatment for DME [yes vs. no]) as fixed factors; and terms for the interaction between baseline BCVA and visit and the interaction between treatment and visit. Missing data were covered by the MMRM. Error bars indicate 95% CIs. *2q8* aflibercept 2 mg every 8 weeks, *8q12* aflibercept 8 mg every 12 weeks, *8q16* aflibercept 8 mg every 16 weeks, *BCVA* best-corrected visual acuity, *CI* confidence interval, *CRT* central retinal thickness, *DME* diabetic macular edema, *LS* least squares, *MMRM* mixed model for repeated measures

Comparable gains in visual acuity were observed in the Japan and non-Japan subgroups (Fig. 1). In the Japan subgroup, the mean (standard deviation [SD]) change from baseline in BCVA at Week 48 was +7.0 (6.6) letters in the 8q12 group, +7.4 (6.8) letters in the 8q16 group, and +8.0 (7.8) letters in the 2q8 group. The LS mean (95% CI) change from baseline in BCVA at Week 48 was +7.1 (4.9 to 9.2), +7.5 (4.4 to 10.7), and +7.4 (3.8 to 11.0) letters in the 8q12, 8q16, and 2q8 groups, respectively. The difference in LS means (95% CI) was -0.30 letters (-4.45 to 3.86) between 8q12 and 2q8 and +0.17 letters (-4.44 to 4.78) between 8q16 and 2q8. In the non-Japan subgroup, the mean (SD) change from baseline in BCVA at Week 48 was +9.0 (9.2) letters in the 8q12 group, +7.9 (8.6) letters in the 8q16 group, and +9.4 (9.2) letters in the 2q8 group. The LS mean (95% CI) change from baseline in BCVA at Week 48 was +8.4 (7.2 to 9.6), +7.3 (5.9 to 8.7), and +9.1 (7.6 to 10.5) letters in the 8q12, 8q16, and 2q8 groups, respectively. The difference in LS means (95% CI) was -0.64 letters (-2.48 to 1.21) between 8q12 and 2q8 and -1.76 letters (-3.74 to 0.22) between 8q16 and 2q8.

### Durability

**Fig. 2:**
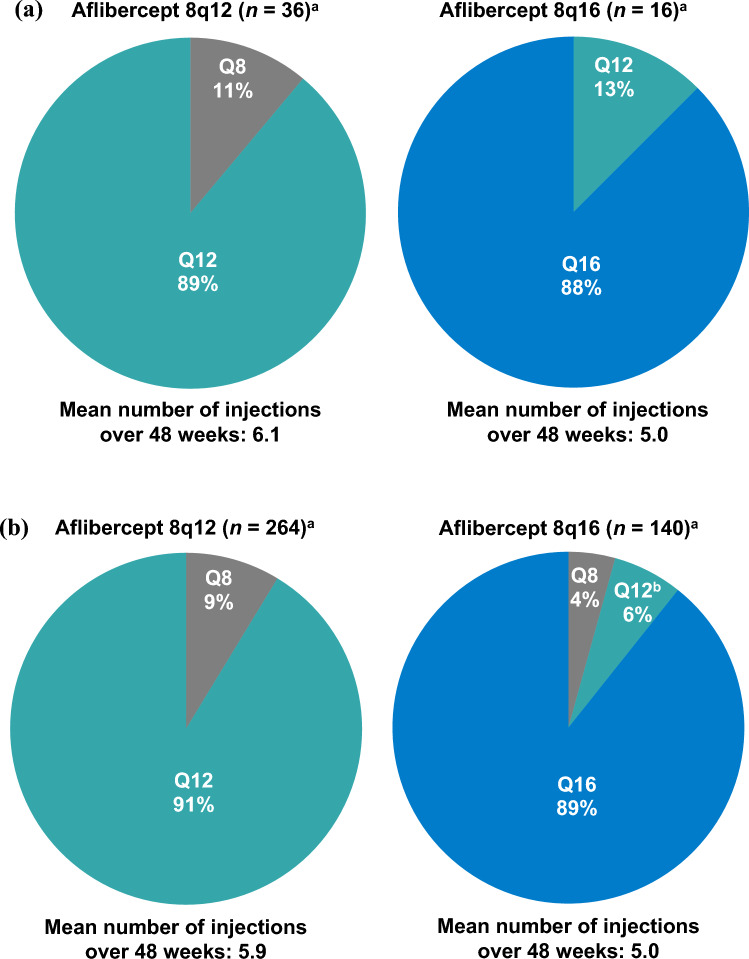
Proportion of aflibercept 8 mg-treated patients who maintained their randomized dosing intervals through Week 48 in the (**a**) Japan and (**b**) non-Japan subgroups of PHOTON. Percentages are based on the number of patients randomized to 8q12 or 8q16 who completed the Week 48 visit segmented by Japan versus non-Japan. Values may not add up to 100% due to rounding. ^a^Patients in the safety analysis set who completed Week 48. ^b^Patients randomly assigned to aflibercept 8q16 whose dosing intervals were shortened to 8q12, excluding those whose dosing intervals were later shortened to aflibercept 8 mg Q8 from 8q12. *8q12* aflibercept 8 mg every 12 weeks, *8q16* aflibercept 8 mg every 16 weeks, *Q8* every 8 weeks, *Q12* every 12 weeks, *Q16* every 16 weeks

Durability (based on participants who had completed Week 48) was comparable between the Japan and non-Japan subgroups. In the Japan and non-Japan subgroups, respectively, 92% (48/52) and 93% (375/404) of patients who received aflibercept 8 mg maintained ≥ 12-week dosing intervals through Week 48. In the Japan subgroup, 89% (32/36) of patients in the 8q12 group and 88% (14/16) of patients in the 8q16 group maintained their randomized dosing intervals through Week 48, while in the non-Japan subgroup, 91% (241/264) of patients in the 8q12 group and 89% (125/140) of patients in the 8q16 group maintained their randomized dosing intervals through Week 48 (Fig. 2).

### DRSS score

**Fig. 3:**
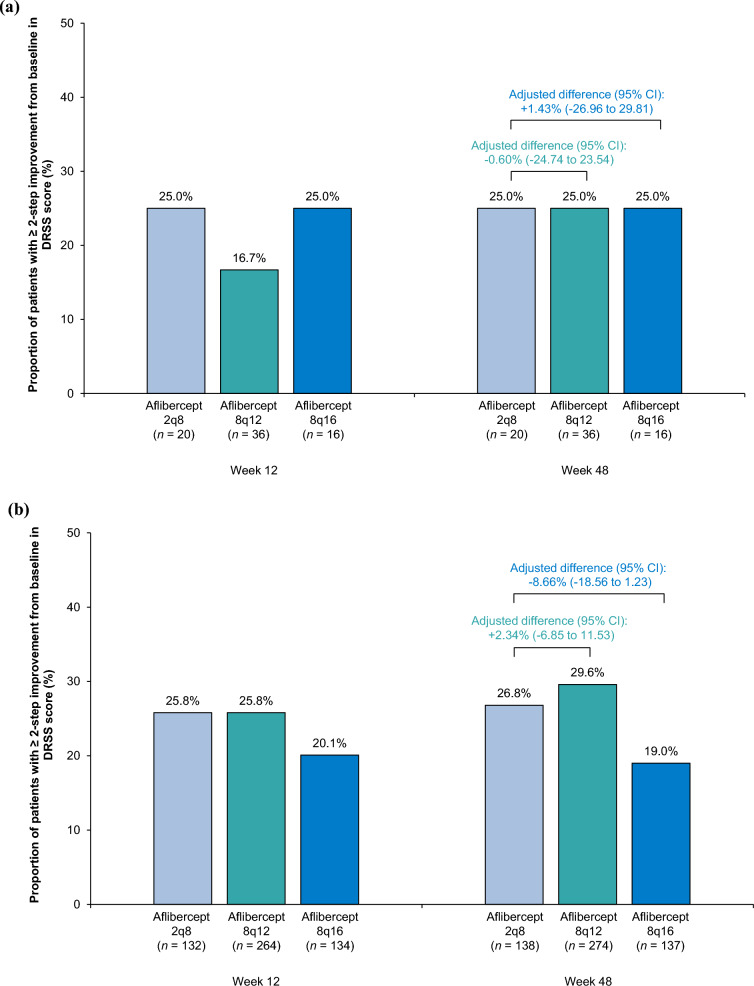
Proportion of patients in the (**a**) Japan and (**b**) non-Japan subgroups of PHOTON with ≥ 2-step DRSS improvement from baseline at Weeks 12 and 48. Full analysis set. The proportion of patients with ≥ 2-step improvement in DRSS score at Weeks 12 and 48 in the Japan (**a**) and the non-Japan (**b**) subgroups. Adjusted difference (95% CI) between treatment groups at Week 48 is shown above the bars. The adjusted difference and 95% CI at Week 48 were calculated using the Mantel-Haenszel weighting scheme adjusted for stratification variables (geographic region [Japan vs. rest of the world], baseline CRT [< 400 μm vs. ≥ 400 μm], previous treatment for DME [yes vs. no]). Missing data and excluded data due to intercurrent events were imputed using the last observation carried forward approach. *2q8* aflibercept 2 mg every 8 weeks, *8q12* aflibercept 8 mg every 12 weeks, *8q16* aflibercept 8 mg every 16 weeks, *CI* confidence interval, *CRT* central retinal thickness, *DME* diabetic macular edema, *DRSS* Diabetic Retinopathy Severity Scale

In the Japan subgroup, the proportion of patients that achieved ≥ 2-step improvement from baseline in DRSS score at Week 48 was 25.0% (9/36) in the 8q12 group, 25.0% (4/16) in the 8q16 group, and 25.0% (5/20) in the 2q8 group (Fig. 3). In the non-Japan subgroup, corresponding proportions were 29.6% (81/274), 19.0% (26/137), and 26.8% (37/138). The adjusted difference (95% CI) in the proportion of patients that achieved ≥ 2-step improvement from baseline in DRSS score between 8q12 and 2q8 was -0.60% (-24.74% to 23.54%) in the Japan subgroup and 2.34% (-6.85% to 11.53%) in the non-Japan subgroup. The adjusted difference (95% CI) between 8q16 and 2q8 was 1.43% (-26.96% to 29.81%) in the Japan subgroup and -8.66% (-18.56% to 1.23%) in the non-Japan subgroup.

### CRT

**Fig. 4:**
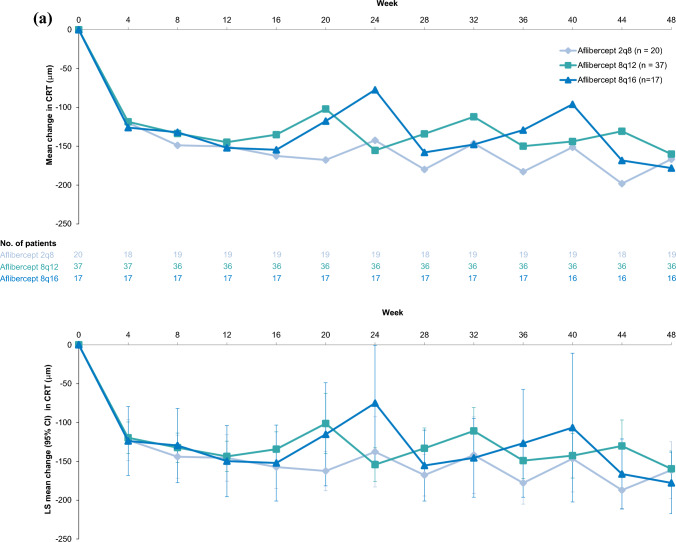

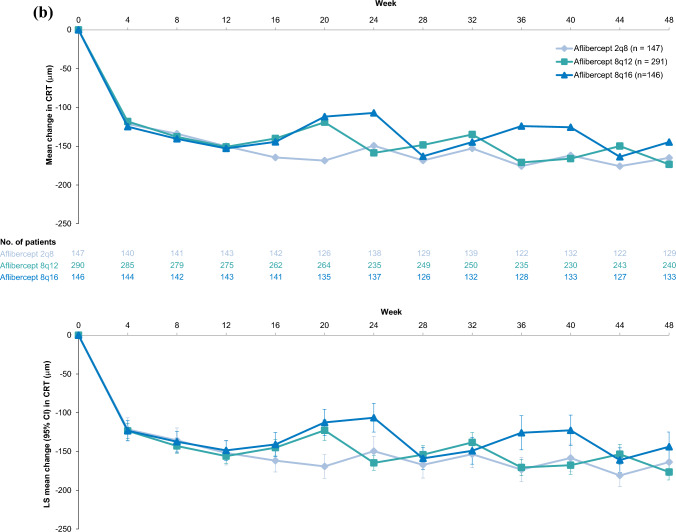
Mean change and LS mean change from baseline in CRT through Week 48 in the (**a**) Japan and (**b**) non-Japan subgroups of PHOTON. Full analysis set. Mean change from baseline in CRT through Week 48 in the Japan (**a**) and the non-Japan (**b**) subgroups are presented in the top panels. Data are arithmetic means of observed data before relevant intercurrent events. The number of patients included in the analysis, after accounting for relevant intercurrent events and missing data, are provided for each timepoint. LS mean change from baseline in CRT at Week 48 in the Japan (**a**) and the non-Japan (**b**) subgroup are presented in the bottom panels. Data are LS means that were generated using MMRM, based on observed data before relevant intercurrent events, with baseline CRT as a covariate; treatment group, visit, stratification variables (geographic region [Japan vs. rest of the world], baseline CRT [< 400 μm vs. ≥ 400 μm], and previous treatment for diabetic macular edema [yes vs. no]) as fixed factors; and terms for the interaction between baseline CRT and visit and the interaction between treatment and visit. Missing data were covered by the MMRM. Retinal thickness measurements were based on optical coherence tomography segmentation from the inner limiting membrane to the retinal pigment epithelium. *2q8* aflibercept 2 mg every 8 weeks, *8q12* aflibercept 8 mg every 12 weeks, *8q16* aflibercept 8 mg every 16 weeks, *CI* confidence interval, *CRT* central retinal thickness, *LS* least squares, *MMRM* mixed model for repeated measures

Comparable improvements in CRT were observed in the Japan and non-Japan subgroups (Fig. 4). In the Japan subgroup, mean (SD) change from baseline in CRT at Week 48 was -160.1 (104.6) μm in the 8q12 group, -178.0 (121.7) μm in the 8q16 group, and -166.6 (142.9) μm in the 2q8 group. The LS mean (95% CI) change from baseline in CRT was -159.6 (-182.5 to -136.6) μm, -177.7 (-217.2 to -138.2) μm, and -161.2 (-197.6 to -124.7) μm in the 8q12, 8q16, and 2q8 groups, respectively. In the non-Japan subgroup, mean (SD) change from baseline in CRT at Week 48 was -173.4 (146.4) μm in the 8q12 group, -144.7 (134.5) μm in the 8q16 group, and -165.1 (140.4) μm in the 2q8 group. The LS mean (95% CI) change from baseline in CRT was -176.4 (-186.7 to -166.2) μm, -143.8 (-162.9 to -124.8) μm, and -163.7 (-181.3 to -146.2) μm in the 8q12, 8q16, and 2q8 groups, respectively.

### Safety

The safety profiles of aflibercept 8 mg and 2 mg were comparable in the Japan and non-Japan subgroups (Table 2). In the Japan subgroup, ocular TEAEs in the study eye were reported in 32.4% (12/37) of patients in the 8q12 group, 35.3% (6/17) of patients in the 8q16 group, and 30.0% (6/20) of patients in the 2q8 group. In the non-Japan subgroup, ocular TEAEs in the study eye were reported in 31.6% (92/291) of patients in the 8q12 group, 28.8% (42/146) of patients in the 8q16 group, and 27.2% (40/147) of patients in the 2q8 group. In the Japan subgroup, the following ocular TEAEs in the study eye were reported in ≥ 5% of patients in any treatment group (Online Resource 3): conjunctival hemorrhage (n = 4); conjunctivitis, corneal erosion, diabetic retinal edema, IOP increased, keratitis, punctate keratitis, and vitreous floaters (n = 2 for each TEAE); and asthenopia, dry eye, epiretinal membrane, IOP decreased, retinal artery stenosis, vision blurred, visual field defect, and vitreous opacities (n = 1 for each TEAE). In the non-Japan subgroup, the ocular TEAEs in the study eye of cataract and vitreous floaters were reported in ≥ 5% of patients in any treatment group. In the Japan subgroup, no serious ocular TEAEs were reported in the study eye. Serious ocular TEAEs in the study eye were reported in 0.7% (2/291) of patients in the 8q12 group, 0.7% (1/146) of patients in the 8q16 group, and 0.7% (1/147) of patients in the 8q12 group within the non-Japan subgroup; serious ocular TEAEs in the study eye were cataract subcapsular, increased IOP, retinal detachment, ulcerative keratitis, and vitreous hemorrhage (n = 1 for each serious ocular TEAE), all of which were considered unrelated to aflibercept treatment. No patients in the Japan subgroup experienced intraocular inflammation (IOI), and no cases of endophthalmitis or retinal vasculitis were reported. In the non-Japan subgroup, the proportion of patients with IOI was low across all treatment groups (8q12: 1.4% [4/291]; 8q16: 0%; 2q8: 0.7% [1/147]), with no endophthalmitis or retinal vasculitis reported. Additionally, mean changes from baseline in pre-dose IOP did not exceed approximately 1 mm Hg at any visit through Week 48 in either subgroup.

**Table 2:** Summary of key adverse events up to Week 48 of PHOTON in the Japan and non-Japan subgroups

	Japan			Non-Japan		
	Aflibercept 2q8 (n = 20)	Aflibercept 8q12 (n = 37)	Aflibercept 8q16 (n = 17)	Aflibercept 2q8 (n = 147)	Aflibercept 8q12 (n = 291)	Aflibercept 8q16 (n = 146)
Patients with ≥ 1 ocular TEAE, n (%)^a^	6 (30.0)	12 (32.4)	6 (35.3)	40 (27.2)	92 (31.6)	42 (28.8)
Patients with ≥ 1 serious ocular TEAE, n (%)^a^	0	0	0	1 (0.7)	2 (0.7)	1 (0.7)
Patients with ≥ 1 non-ocular TEAE, n (%)	12 (60.0)	19 (51.4)	11 (64.7)	67 (45.6)	155 (53.3)	84 (57.5)
Patients with ≥ 1 serious non-ocular TEAE, n (%)	1 (5.0)	2 (5.4)	2 (11.8)	25 (17.0)	50 (17.2)	20 (13.7)
Patients with ≥ 1 treatment-related ocular TEAE, n (%)^a^	2 (10.0)	0	0	1 (0.7)	5 (1.7)	0
Patients with ≥ 1 intraocular inflammation event, n (%)^a^	0	0	0	1 (0.7)	4 (1.4)	0
Iridocyclitis	0	0	0	1 (0.7)	0	0
Iritis	0	0	0	0	1 (0.3)	0
Uveitis	0	0	0	0	1 (0.3)	0
Vitreal cells	0	0	0	0	1 (0.3)	0
Vitritis	0	0	0	0	1 (0.3)	0
Patients with intraocular pressure ≥ 35 mm Hg pre-injection or post-injection, n (%)^a^	1 (5.0)	1 (2.7)	0	1 (0.7)	0	0
Patients with ≥ 1 treatment-emergent APTC event, n (%)^b^	0	0	0	6 (4.1)	8 (2.7)	7 (4.8)
Non-fatal myocardial infarction	0	0	0	3 (2.0)	3 (1.0)	1 (0.7)
Non-fatal stroke	0	0	0	0	3 (1.0)	5 (3.4)
Vascular death	0	0	0	3 (2.0)	2 (0.7)	1 (0.7)
Treatment-emergent hypertension, n (%)	4 (20.0)	1 (2.7)	1 (5.9)	16 (10.9)	35 (12.0)	22 (15.1)
Death, n (%)	0	0	0	4 (2.7)	9 (3.1)	3 (2.1)

Safety analysis set. Percentages are based on n values provided in column headings

*2q8* aflibercept 2 mg every 8 weeks, *8q12* aflibercept 8 mg every 12 weeks, *8q16* aflibercept 8 mg every 16 weeks, *APTC* Antiplatelet Trialists’ Collaboration, *TEAE* treatment-emergent adverse event

^a^Reported in the study eye

^b^Adjudicated by a masked committee

In the Japan subgroup, non-ocular TEAEs were reported in 51.4% (19/37) of patients in the 8q12 group, 64.7% (11/17) of patients in the 8q16 group, and 60.0% (12/20) of patients in the 2q8 group. In the non-Japan subgroup, non-ocular TEAEs were reported in 53.3% (155/291) of patients in the 8q12 group, 57.5% (84/146) of patients in the 8q16 group, and 45.6% (67/147) of patients in the 2q8 group. In the Japan subgroup, serious non-ocular TEAEs were reported in 5.4% (2/37) of patients in the 8q12 group, 11.8% (2/17) of patients in the 8q16 group, and 5.0% (1/20) of patients in the 2q8 group. In the non-Japan subgroup, serious non-ocular TEAEs were reported in 17.2% (50/291) of patients in the 8q12 group, 13.7% (20/146) of patients in the 8q16 group, and 17.0% (25/147) of patients in the 2q8 group. There were no treatment-emergent Antiplatelet Trialists’ Collaboration (APTC)-defined arterial thromboembolic events reported in the Japan subgroup. In the non-Japan subgroup, treatment-emergent APTC thromboembolic events were reported in 2.7% (8/291) of patients in the 8q12 group, 4.8% (7/146) of patients in the 8q16 group, and 4.1% (6/147) of patients in the 2q8 group. In the Japan subgroup, treatment-emergent hypertension was reported in 2.7% (1/37) of patients in the 8q12 group, 5.9% (1/17) of patients in the 8q16 group, and 20.0% (4/20) of patients in the 2q8 group. In the non-Japan subgroup, treatment-emergent hypertension was reported in 12.0% (35/291) of patients in the 8q12 group, 15.1% (22/146) of patients in the 8q16 group, and 10.9% (16/147) of patients in the 2q8 group. There were no deaths in the Japan subgroup. In the non-Japan subgroup, death was reported in 2.7% (16/584) of patients by Week 48 (8q12: 3.1% [9/291]; 8q16: 2.1% [3/146]; 2q8: 2.7% [4/147]). All deaths were deemed unrelated to aflibercept treatment.

## Discussion

This is the first analysis to evaluate the efficacy, durability, and safety of aflibercept 8 mg in Japanese patients with DME. Consistent with the findings from the primary analysis of the PHOTON trial [24], patients in the Japan and non-Japan subgroups who were treated with aflibercept 8 mg achieved comparable BCVA gains to aflibercept 2 mg at Week 48 with fewer injections. In addition, the proportion of patients in the Japan and non-Japan subgroups achieving ≥ 2-step improvement from baseline in DRSS score at Week 48 was consistent with that of the overall population in the PHOTON trial [24]. However, these findings could potentially be confounded by the small number of patients in the Japan subgroup.

Despite fewer injections, comparable anatomic improvements were observed with aflibercept 8 mg and 2 mg in the Japan and non-Japan subgroups. CRT reductions were similar across all treatment groups and were comparable to those of the overall PHOTON patient population [24].

Consistent with the overall PHOTON patient population [24], approximately 90% of patients who were randomized to aflibercept 8q12 or 8q16 and completed Week 48 in the Japan and non-Japan subgroups maintained their randomized dosing intervals through Week 48 without the need for interval shortening. In analyses of real-world data, Japanese patients with DME who were treated with aflibercept 2 mg experienced worse visual and anatomic outcomes versus those reported in the VISTA and VIVID trials, in part due to fewer injections and, therefore, potential undertreatment [28]. In the current analysis, similar visual and anatomic improvements to aflibercept 2 mg were observed with extended dosing intervals in Japanese patients treated with aflibercept 8 mg, suggesting that disease was adequately controlled with aflibercept 8 mg at 12- and 16-week intervals following three initial monthly doses in this patient population.

Aflibercept 8 mg demonstrated a similar safety profile to that of aflibercept 2 mg in both the Japan and non-Japan subgroups, consistent with observations in the overall PHOTON patient population [24]. Similarly, no new safety signals with aflibercept 8-mg treatment were identified in either subgroup. No events of IOI were reported in patients within the Japan subgroup, and no clinically relevant changes in IOP were reported. Furthermore, as no cases of endophthalmitis or retinal vasculitis were reported in the overall PHOTON patient population [24], no cases were reported in either subgroup.

This subgroup analysis of the PHOTON trial has several limitations. Due to the small sample size in the Japan subgroup, findings should be interpreted with caution. Inherent limitations of the PHOTON study design also apply [24] given that direct comparisons of treatment effects were not readily feasible due to asynchronous dosing across the aflibercept 8q12, 8q16, and 2q8 groups after Week 8, and the 48-week analysis limits efficacy evaluations of aflibercept 8 mg to 1 year. An analysis of 96-week data from the overall PHOTON population suggests that patients treated with aflibercept 8 mg maintain long-term visual and anatomic improvements with extended dosing intervals [29]. Future sub-analyses in Japanese patients from the PHOTON trial treated beyond 1 year, as well as observational and real-world analyses, will be important to determine whether visual and anatomic improvements are maintained long term with extended dosing in Japanese patients.

In conclusion, this subgroup analysis of Japanese and non-Japanese patients with DME from the PHOTON trial shows that visual and anatomic improvements with aflibercept 8q12 and 8q16 were generally comparable to those with aflibercept 2q8 through Week 48. These improvements with aflibercept 8 mg were achieved with extended dosing intervals and fewer injections than aflibercept 2 mg in both subgroups. These results are consistent with those of the overall PHOTON patient population, providing support for improved treatment outcomes and reduced treatment burden with aflibercept 8 mg in Japanese patients with DME.

## Supplementary Information

Below is the link to the electronic supplementary material.

Supplementary file1 (PDF 12 KB)

Supplementary file2 (PDF 134 KB)

Supplementary file3 (PDF 162 KB)

## Data Availability

Qualified researchers may request access to study documents (including the clinical study report, study protocol with any amendments, blank case report form, statistical analysis plan) that support the methods and findings reported in this manuscript. Individual anonymized participant data will be considered for sharing 1) once the product and indication has been approved by major health authorities (e.g., FDA, EMA, PMDA, etc.) or development of the product has been discontinued globally for all indications on or after April 2020 and there are no plans for future development 2) if there is legal authority to share the data and 3) there is not a reasonable likelihood of participant re-identification. Submit requests to https://vivli.org/.
