# Supplementary material for: Intravitreal aflibercept 8 mg in patients from Japan with diabetic macular edema: 48-week subgroup analysis of the PHOTON trial
Source: Jpn J Ophthalmol. 2025 Dec 26;70(1):123–38. doi: 10.1007/s10384-025-01271-7 (PMC12948817; doi:10.1007/s10384-025-01271-7)
Supplement: Supplementary file 1 — Supplementary file1 (PDF 12 KB) [file 10384_2025_1271_MOESM1_ESM.pdf]

Online Resource 1. List of Institutional Review Boards and Ethics Committees.

Advarra; Health Research Ethics Board of Alberta; Eticka komise Vseobecne fakultni nemocnice v Praze; Eticka komise Fakultni nemocnice Kralovske Vinohrady; Eticka komise Fakultni nemocnice Hradec Kralove; Eticka komise Axon Clinical; Ethikkommission der Universitatsmedizen Gottingen; Ethikkommission der Medizinischen Fakultät der Universität zu Lübeck; Ethikkommission des Klinikums der Philipps Universität Marburg; Ethikkommission der Medizinischen Fakultät der Universität Würzburg; Ethikkommission zur Beurteilung medizinischer Forschung am Menschen der Ärztekammer Niedersachsen; Ethikkommission an der TU Dresden; Medical Research Council Ethics Committee for Clinical Pharmacology; Institutional Review Board of Hayashi Eye Hospital; Mie University Hospital Institutional Review Board; Nagasaki University Institutional Review Board; Tokyo Medical University Hachioji Medical Center Institutional Review Board; IRB of Kobori Clinic; Kagoshima University Hospital Institutional Review Board; Shinsu University Hospital Institutional Review Board; Osaka Metropolitan University Hospital Institutional Review Board; Yamaguchi University Hospital Institutional Review Board; Japanese Red Cross Saitama Hospital Institutional Review Board; Kansai Medical University Hospital Institutional Review Board; Nagoya City University Institutional Review Board; St. Marianna University Group Institutional Review Board; Nihon University Hospitals Joint Institutional Review Board; University of Fukui Hospital Institutional Review Board; Tokushima University Hospital Institutional Review; Nara Medical University Hospital Institutional Review; National Defense Medical College Hospital Institutional Review Board; Nagoya University Hospital Institutional Review Board; National Hospital Organization Tokyo Medical Center Institutional Review Board; Southern Tohoku General Hospital Institutional Review Board; Jichi Medical University Hospital Institutional

Review Board; Aichi Medical University Hospital Institutional Review Board; Kozawa Eye Hospital and Diabetes Center Institutional Review Board; Kagawa University Hospital Institutional Review Board; Tokyo Metropolitan Geriatric Hospital Institutional Review Board; Kobe University Hospital Institutional Review Board; Joint Institutional Review Board; Shintokai Yokohama Minoru Clinic Institutional Review Board; Local Ethics Committee of Private Healthcare Institution "Clinical Hospital" Russian Railways - Medicine" of the city of Saratov"; Institutional Review Board South Central - Oxford B Research Ethics Committee; University of Oklahoma Health Sciences Center Institutional Review Board; Kaiser Permanente Southern California Institutional Review Board; Stanford University.
