# Supplementary material for: Intravitreal aflibercept 8 mg in patients from Japan with diabetic macular edema: 48-week subgroup analysis of the PHOTON trial
Source: Jpn J Ophthalmol. 2025 Dec 26;70(1):123–38. doi: 10.1007/s10384-025-01271-7 (PMC12948817; doi:10.1007/s10384-025-01271-7)
Supplement: Supplementary file 2 — Supplementary file2 (PDF 134 KB) [file 10384_2025_1271_MOESM2_ESM.pdf]

Online Resource 2. Patient disposition in the Japan and non-Japan subgroups of PHOTON.

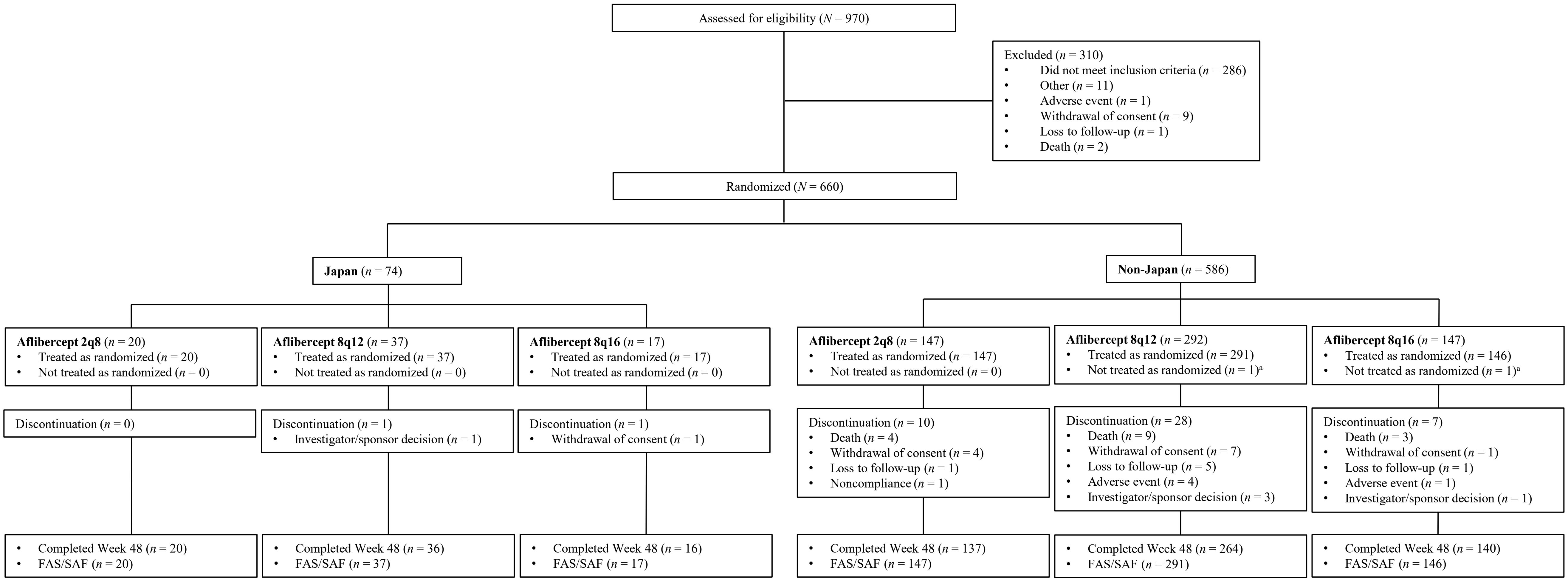

[a]One patient was randomly assigned in error and did not receive treatment. *2q8* aflibercept 2 mg every 8 weeks, *8q12* aflibercept 8 mg every 12 weeks, *8q16* aflibercept 8 mg every 16 weeks, *FAS* full analysis set, *SAF* safety analysis set
