# Supplementary material for: Intravitreal aflibercept 8 mg in patients from Japan with diabetic macular edema: 48-week subgroup analysis of the PHOTON trial
Source: Jpn J Ophthalmol. 2025 Dec 26;70(1):123–38. doi: 10.1007/s10384-025-01271-7 (PMC12948817; doi:10.1007/s10384-025-01271-7)
Supplement: Supplementary file 3 — Supplementary file3 (PDF 162 KB) [file 10384_2025_1271_MOESM3_ESM.pdf]

Online Resource 3. Ocular TEAEs reported in the study eye of ≥ 5% of patients in any treatment group in the Japan and non-Japan subgroups of PHOTON.

	Japan			Non-Japan		
	Aflibercept 2q8 (*n* = 20)	Aflibercept 8q12 (*n* = 37)	Aflibercept 8q16 (*n* = 17)	Aflibercept 2q8 (*n* = 147)	Aflibercept 8q12 (*n* = 291)	Aflibercept 8q16 (*n* = 146)
Cataract[a]	0	0	0	5 (3.4)	11 (3.8)	10 (6.8)
Conjunctival hemorrhage	0	3 (8.1)	1 (5.9)	6 (4.1)	11 (3.8)	5 (3.4)
Punctate keratitis	0	1 (2.7)	1 (5.9)	1 (0.7)	4 (1.4)	5 (3.4)
Vitreous floaters	1 (5.0%)	1 (2.7)	0	3 (2.0)	15 (5.2)	3 (2.1)
Diabetic retinal edema	1 (5.0%)	0	1 (5.9)	2 (1.4)	9 (3.1)	2 (1.4)

Vision blurred	1 (5.0)	0	0	2 (1.4)	4 (1.4)	2 (1.4)
Dry eye	0	0	1 (5.9)	1 (0.7)	2 (0.7%)	1 (0.7)
Intraocular pressure increased	1 (5.0)	1 (2.7)	0	5 (3.4)	6 (2.1)	1 (0.7)
Asthenopia	1 (5.0)	0	0	0	0	0
Conjunctivitis	0	0	2 (11.8)	0	0	0
Corneal erosion	0	0	2 (11.8)	0	0	0
Epiretinal membrane	1 (5.0)	0	0	1 (0.7)	1 (0.3)	0
Intraocular pressure decreased	1 (5.0)	0	0	0	0	0
Keratitis	0	0	2 (11.8)	0	0	0
Retinal artery stenosis	1 (5.0)	0	0	0	0	0
Visual field defect	0	0	1 (5.9)	0	0	0

Vitreous opacities	1 (5.0)	0	0	0	0	0

Safety analysis set. Data are *n* (%). Percentages are based on the total number of patients (*n* values) provided in the column headings

2q8 aflibercept 2 mg every 8 weeks, *8q12* aflibercept 8 mg every 12 weeks, *8q16* aflibercept 8 mg every 16 weeks, *TEAE* treatment-emergent adverse event

[a]The following preferred terms related to cataract were identified and summarized under the grouped term cataract: cataract, cataract cortical, cataract nuclear, cataract operation complication, cataract subcapsular, and lenticular opacities
